# Public Attitudes Toward Mental Health Treatment Policy

**DOI:** 10.1001/jamanetworkopen.2025.32344

**Published:** 2025-09-17

**Authors:** Morgan C. Shields, Nev Jones, Shyamal Sharma, Susan H. Busch

**Affiliations:** 1 School of Public Health, Washington University in St Louis, St Louis, Missouri; 2 School of Social Work, University of Pittsburgh, Pittsburgh, Pennsylvania; 3 Heller School for Social Policy and Management, Brandeis University, Waltham, Massachusetts; 4 School of Public Health, Yale University, New Haven, Connecticut

## Abstract

This cross-sectional study examines public attitudes toward mental health care policies, including community-based, peer-led, and involuntary services, by political party affiliation.

## Introduction

Rates of mental health conditions are increasing, yet only about one-half of people with a mental health condition and less than one-quarter with a substance use disorder (SUD) received treatment in 2023.^1^ In response, policies have aimed to increase access to mental health care, including crisis services.^2,3,4^ One tension relates to support for community-based services vs increasing emphasis on involuntary interventions.^5^ To inform decision-making, it is important to understand public attitudes toward these policy choices.

## Methods

This cross-sectional study was approved by the institutional review board at Washington University in St Louis. We followed the STROBE reporting guidelines for cross-sectional studies. We conducted a national internet-based survey of US adults (January 17 to February 12, 2025) through Qualtrics using quota sampling on gender, age, race, ethnicity, income, education, and region to reflect census population estimates. Participants provided consent electronically through the survey. Participants were asked to indicate their support for expansions in broad mental health care policies on a 9-point Likert scale, including community-based services, peer-led services, and involuntary services; we created binary variables from responses that capture support for those with values 6 to 9 on the scale (see the eAppendix in Supplement 1 for survey details).

To examine differences by party, we asked people what political party they identify with. We used χ^2^ tests to examine unadjusted differences between Democrats and Republicans and linear probability regression models to examine adjusted differences across parties, reporting statistically significant differences at 2-tailed α = .05. Descriptive statistics were weighted on the characteristics used in quota sampling to account for remaining differences in representation, as well as political party affiliation; these same variables served as controls in regressions, with the addition of rurality and history of using behavioral health services. Data were analyzed with Stata statistical software version 18 (StataCorp).

## Results

Of 1442 participants, 849 (58.9%) were female, and 292 (20.3%) had annual household income less than $25 000. In unadjusted weighted estimates, 72.64% (95% CI, 68.81% to 76.47%) supported policies to expand access to community services, and most participants supported policies to expand access to peer-led services (65.10%; 95% CI, 61.09% to 69.11%) (Table). We found no evidence that levels of support differed between Republicans and Democrats. These trends persisted in adjusted models (Figure).

**Table.** Support for Mental Health Policies Overall and Across Political Party, Unadjusted Weighted

Items	Respondents, No. (%) [95% CI]					Republican vs Democrat^a^	
	Overall (N = 1442)	Republican (n = 524)	Democrat (n = 495)	Independent (n = 339)	Other (n = 84)	Difference^b^	*P* value^c^
Noncoercive policies							
Expand access to community-based mental health care services	1047 (72.64) [68.81 to 76.47]	377 (71.94) [65.19 to 78.69]	385 (77.85) [72.40 to 83.30]	240 (70.75) [62.57 to 78.93]	45 (53.80) [36.59 to 71.00]	−5.91	.18
Expand access to peer-led services	939 (65.10) [61.09 to 69.11]	369 (70.34) [63.78 to 76.90]	341 (68.84) [62.96 to 74.72]	186 (54.87) [45.89 to 63.84]	42 (49.47) [32.54 to 66.39]	1.50	.74
Coercive policies							
Make it easier to force a person to take psychiatric medication against their will even when they have not committed a crime	575 (39.90) [35.71 to 44.09]	237 (45.27) [37.71 to 52.82]	228 (46.13) [39.79 to 52.48]	92 (27.21) [19.41 to 35.01]	16 (18.81) [7.26 to 30.37]	−0.86	.86
Make it easier to force a person to be hospitalized in a psychiatric facility against their will for short-term care	650 (45.05) [40.88 to 49.21]	285 (54.32) [46.87 to 61.78]	221 (44.60) [38.30 to 50.90]	121 (35.77) [27.29 to 44.25]	18 (21.16) [9.80 to 32.53]	9.72	.05
Make it easier to force a person to be hospitalized in a psychiatric facility against their will for long-term care	610 (42.32) [38.21 to 46.43]	264 (50.43) [42.99 to 57.88]	213 (43.13) [36.85 to 49.40]	111 (32.78) [24.85 to 40.72]	17 (20.41) [8.00 to 32.82]	7.30	.14
Make it easier to force a person with a substance use disorder to receive treatment against their will	765 (53.07) [48.91 to 57.23]	320 (61.12) [54.06 to 68.19]	261 (52.64) [46.31 to 58.97]	148 (43.79) [34.94 to 52.65]	32 (38.19) [20.99 to 55.39]	8.48	.08

^a^ Corrected χ^2^ statistics were used to test differences in proportions between Republicans and Democrats, accounting for survey weights.

^b^ Difference in percentage points between Republicans and Democrats.

^c^ *P* value is 2 tailed.

**Figure.**
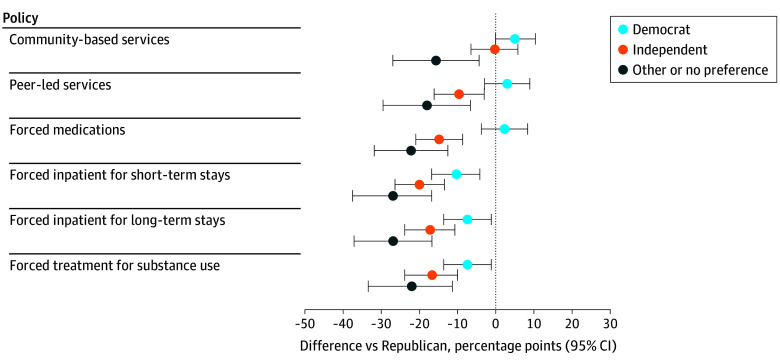
Adjusted Regression Coefficients From Linear Probability Models of Differences in Support for Policies vs Republicans All linear probability models controlled for the following categorical variables: region, rurality, income, education, age, gender, race, ethnicity, prior experience receiving mental health or substance use treatment and used robust SEs to account for potential heteroskedasticity.

In contrast, fewer participants supported policies that make it easier to force a person against their will to take medication (39.90%; 95% CI, 35.71% to 44.09%), be hospitalized for a short-term stay (45.05%; 95% CI 40.88% to 49.21%), be hospitalized for a long-term stay (42.32%; 95% CI, 38.21% to 46.43%), or to receive SUD treatment (53.07%; 95% CI, 48.91% to 57.23%) (Table). There were no statistically significant differences between Republicans and Democrats in unadjusted analyses. In adjusted analyses, Democrats were less supportive of involuntary inpatient care for short-term stays (−10.4%; 95% CI, −16.7% to −4.0%), inpatient care for long-term stays (−7.4%; 95% CI, −13.7% to −1.1%), and SUD treatment (−7.3%; 95% CI, −13.7% to 0.9%) (Figure). Independents and other party affiliation consistently reported lower support for all policies in both unadjusted and adjusted analyses. The full regression models are available from the authors upon request.

## Discussion

National policy has vacillated over time in its focus on involuntary and community-based interventions^5,6^; it remains unclear how these priorities will evolve. The findings of this cross-sectional study indicate that the public largely supports policies that expand voluntary, community-based services, a position shared by members of all political parties. In comparison, the public is less supportive of expansions in involuntary policies, although Republicans report more support than others. As with all surveys using quota sampling, we cannot eliminate potential biases inherent in these surveys. Community-based services are evidence-based ways to improve population health and reduce reliance on costly institutional care. During public polarization on health policy issues, strong bipartisan support and empirical evidence suggests that these policies are politically viable.

## Notes

Supplement 1. **eAppendix.** Supplemental Methods

Supplement 2.
Data Sharing Statement
