# Error in Discussion

**DOI:** 10.1001/jamanetworkopen.2025.52015

**Published:** 2025-12-12

In the Research Letter titled “Public Attitudes Toward Mental Health Treatment Policy,”^1^ published September 17, 2025, there was an error in the Discussion. The corrected sentence states, “In comparison, the public is less supportive of expansions in involuntary policies, although Republicans report more support than others.” In the original article, the phrase *expansions in* was omitted. This article has been corrected.^1^

## References

[1] Shields MC, Jones N, Sharma S, Busch SH. Public attitudes toward mental health treatment policy. JAMA Netw Open. 2025;8(9):e2532344. doi:10.1001/jamanetworkopen.2025.32344 40960830 PMC12444547

