# Supplementary material for: Public Attitudes Toward Mental Health Treatment Policy
Source: JAMA Netw Open. 2025 Sep 17;8(9):e2532344. doi: 10.1001/jamanetworkopen.2025.32344 (PMC12444547; doi:10.1001/jamanetworkopen.2025.32344)
Supplement: Supplement 1. — eAppendix. Supplemental Methods [file jamanetwopen-e2532344-s001.pdf]

Supplemental Online Content

Shields MC, Jones N, Sharma S, Busch SH. Public attitudes toward mental health treatment policy. *JAMA Netw Open*. 2025;8(9):e2532344. doi:10.1001/jamanetworkopen.2025.32344

eAppendix. Supplemental Methods

This supplemental material has been provided by the authors to give readers additional information about their work.

© 2025 Shields MC et al. *JAMA Network Open*.

eAppendix. Supplemental Methods

Survey Design

The overall survey was developed by the research team and took approximately 20 minutes to complete. Participants to the survey were recruited by Qualtrics, which is a commercial survey company that has access to online participant pools, using quota sampling to approximate a nationally representative sample. Prior to launching the survey, Qualtrics engaged in a rigorous process of testing the survey and its logic before fully launching the survey, which included setting up the technical integration and testing it with strategic online panel partners. Qualtrics invited participants in exchange for financial incentives. While Qualtrics managed all recruitment, participants had access to information about the study team, including contact information for our Institutional Review Board.

Variables

Items in the larger survey spanned demographic characteristics, prior experiences with behavioral healthcare services and exposure to people with behavioral health conditions, trust in institutions and professions, attitudes towards behavioral health policies, sources of news, and political orientation.

Demographics

For the current study, our analyses used specific items from the larger survey. These included key demographic characteristics measured in categories: age (18-24, 25-34, 35-44, 45-54, 55-64), gender (male, female, other/nonbinary), race (White, Black or African American, American Indian or Alaskan Native, Asian, Other, More than one race), Ethnicity (Latino/Hispanic), highest level of education (high school or less, some college or associates degree, four-year college degree, graduate school), income (less than $25,000, $25,000-$49,999, $50,000-$74,999, $75,000-$99,999, $100,000 or more), and region (Northeast, Midwest, South, West).

Independent Variables

The main survey asked several questions related to political ideology. We used two of these items. The main item used in all analyses was: *"Generally speaking, do you usually think of yourself as a Republican, a Democrat, an Independent, or something else?"* Response options included Republican, Democrat, Independent, other, and no preference (other and no preference were combined into a category for analyses).

We also asked participants if they voted in the last election (yes/no). We used this item in our supplementary analyses, restricting analyses to those who voted in the 2024 presidential election.

© 2025 Shields MC et al. *JAMA Network Open*.

Dependent Variables

There were six dependent variables that capture support for various types of policies. Below is how these questions were asked:

On a scale of 1-9, please indicate your support for the following mental health and substance use laws or policies

	**Little support** **Strong support**<br>1 2 3 4 5 6 7 8 9
Policies that make it easier to force a person to take psychiatric medication against their will even when they have not committed a crime. ()	
Policies that make it easier to force a person to be hospitalized in a psychiatric facility against their will for **short-term** care. ()	
Policies that make it easier to force a person to be hospitalized in a psychiatric facility against their will for **long-term** care. ()	
Policies that make it easier to force a person with a substance use disorder to receive treatment against their will. ()	
Policies that expand access to community-based mental healthcare services. ()	
Policies that expand access to peer-led services, where people providing mental healthcare are not necessarily formal clinicians but are people who have shared experiences with patients. ()	

For analyses, we created binary variables that indicate “support” for these policies, which we operationalized as any response in the 6-9 range (given that the 5 value is the middle value).

Data Cleaning

Once quotas were reached, Qualtrics cleaned the data for fraudulent responses and bots. This process is described in the following steps (the below list comes directly from documentation provided by Qualtrics Research Services):

1. **Preventing Duplicates**

   Qualtrics has two ways of identifying and preventing duplicate responses, ensuring an individual only completes your survey one time.

© 2025 Shields MC et al. *JAMA Network Open*.

- **The Prevent Ballot Box Stuffing** feature helps to keep respondents from taking a survey multiple times by placing a cookie on their browser when they submit a response. The next time the respondent clicks on the survey link, Qualtrics will see this cookie and prevent them from taking the survey again.

- **RelevantID** is similar to "Prevent Ballot Box Stuffing," but uses enhanced technology to detect duplicates by assessing a respondent's metadata (browser, operating system, and location). The data gathered is put through deterministic algorithms to create a unique digital fingerprint of each computer. The digital fingerprint identifies duplicate respondents who take the same survey more than once from the same machine. RelevantID flags a computer each time a user tries to take a survey, so it is able to detect if multiple email accounts are being used to take surveys from a single computer. In addition, RelevantID has the unique ability to identify multiple panel accounts from different research firms on the same computer. Suspect respondents are flagged in the system and redirected or completely filtered out of surveys in which they attempt to participate. The process is invisible to the user and does not interfere with the user experience. RelevantID is consistent with privacy and data protection laws.

2. **GeoIP Restriction (Outside of Target Country)**

   Qualtrics uses GeoIP (internet geolocation software) to ensure that the survey respondents are coming only from the country or countries you specify.

3. **Speeding**

   Respondents who complete the survey too quickly will be flagged as speeders. This implies they chose answers at random to get through the survey quickly, instead of providing quality answers. Since respondents on mobile devices tend to take longer on average than respondents on computers, speed can be calculated separately by device. The standard threshold for determining a speeder is anyone who completes the survey in less than 1/2 the median time.

4. **Bot Detection**

   Bot detection uses Google's invisible reCaptcha technology to track which responses are likely bots by adding a field to each response called Q _ RecaptchaScore. Every response is rated on the probability that the respondent is a bot. Although this check is very good at identifying bots, it can also lead to false positives (i.e., it may flag some real respondents as bots). Therefore, rather than immediately filtering out these responses, Qualtrics will flag them as suspicious and will review the survey responses with investigators (especially the open-ended responses) to determine whether the response is a bot or not.

5. **Open-Ended Responses**

   The Qualtrics Data Quality Team reviews open-ended survey responses and flags suspicious open-ended responses. They then review these responses to determine whether the responses are valid and should be kept or whether the responses should be removed. The data quality team will flag the following types of responses.

- Gibberish. Respondents who enter ambiguous text into text entry fields will be flagged. Ambiguous text includes “gibberish, ”or cases where the respondent typed random letters and / or symbols to respond to a question.
- Nonsensical. Respondents who enter nonsensical text or provide responses that do not match the question will be flagged. These responses suggest bots or respondents who are not paying sufficient attention to the survey.
- Repetitive. Respondents who enter the same response to multiple open-ended text entry fields will be flagged. This suggests that the respondent is either cutting and pasting or providing the same response throughout in order to move through the survey more quickly.
- Profane. Respondents who enter profanity into text entry fields will be flagged. Care is given to profanity because it does not always indicate fraud or bots.

6. **Contradictory Answers**

   Qualtrics performs a check for any inconsistent or contradictory responses across survey items – for example a respondent who indicated they are 19 years old and retired.

7. **Straightliners**

   Straightlining is the practice of providing the same answers on matrix tables (grids) to quickly get through the questions. For example, a respondent may always choose the first answer to every statement, regardless of how they feel. For many questions, it is reasonable for respondents to provide the same answer across all items in a grid. As a result, the standard for the Qualtrics data quality team is to check for straightlining on 3 or more matrix tables in a survey.

8. **Selecting All Options on a Multi-Select Question**

   Respondents who select all or most responses on check-all-that-apply questions—for example, indicating they have every type of disease, car, or phone listed in the question-- are likely falsifying their response in an attempt to qualify for a survey.

© 2025 Shields MC et al. *JAMA Network Open*.
