# Supplementary material for: Public Attitudes Toward Mental Health Treatment Policy
Source: JAMA Netw Open. 2025 Sep 17;8(9):e2532344. doi: 10.1001/jamanetworkopen.2025.32344 (PMC12444547; doi:10.1001/jamanetworkopen.2025.32344)
Supplement: Supplement 2. — Data Sharing Statement [file jamanetwopen-e2532344-s002.pdf]

Data Sharing Statement

Shields. Public Attitudes Toward Mental Health Treatment Policy. *JAMA Netw Open*. Published September 17, 2025. doi:10.1001/jamanetworkopen.2025.32344

Data
Data available: No
